# Clinican Scientists? Medical Scientists? Clinician and Medical Science Educators!

**DOI:** 10.3205/zma001077

**Published:** 2016-11-15

**Authors:** Martin R. Fischer, Götz Fabry

**Affiliations:** 1 Klinikum der Universität München, Institut für Didaktik und Ausbildungsforschung in der Medizin, München, Germany; 2 GMS Journal for Medical Education, Chief Editor, Erlangen, Germany; 3 Albert-Ludwig-Universität Freiburg, Abt. für Med. Psychologie, Freiburg/Brg, Germany; 4 GMS Journal for Medical Education, Assistant Chief Editor, Erlangen, Germany

## Editorial

The university medicine in Germany is indispensable. There is no doubt about that. In its statement on the “Perspective of University Medicine” [1] published on October 21, 2016, the Science Council (“Wissenschaftsrat”) rightly stated that university medicine is the interface between the science and health system "with its singular task of research, teaching and healthcare ( p. 17).

Patients should be treated as best as possible according to the most up-to-date rules of medical practice. To this end, innovation is needed, which results from research only. But what is also needed and indispensable is teaching to pass on the new knowledge. And to create a common foundation of knowledge, skills and attitudes, so that relevant research questions can arise to improve health care. Translational research from the bench to the bedside and back. An important cycle that runs the risk of stagnation since basic medical researchers are generally no physicians anymore and clinicians are no longer doing enough research.

Without a translational research that connects basic medicine with clinical medicine, the central functions of the university medicine cannot be sustained, according to the scientific council: “Scientifically and clinically active university physicians need reliable scope for development and conditions, that allows them to work on specific subject-related as well as interdisciplinary research questions within the clinical context. Only these prerequisites allow to unlock the specific potentials of university medicine for translation, i.e. the necessary interactions needed to transfer basic research into clinical application and the health care system”(p. 16) [1].

However, as academic career paths seem to become increasingly unattractive for organizational and financial reasons, the development of differentiated research-oriented career paths seems inevitable. Thus, the Scientific Council calls for the establishment of a “Clinician-Scientist” career path for 5% to 8% of the residents working at university hospitals, as has also been demanded by the DFG's permanent Senate Commission for a long time [2]. In addition, better and more sustainable career options in university medicine should be offered for the non-medical researchers in the basic science departments as “medical scientists”. Besides the vague career perspectives other than a professorship, the inferior compensation in comparison to doctors is a problem here. These are clear proposals for a secure academic future which will not be easy to implement in light of the high financial pressure under which university hospitals operate but which are well-founded and reasonable. Without research there are no improvements in patient care. Without young scientists who are motivated by research, with appropriate career perspectives, there is no sustainable university medicine.

And what about teaching in this regard? Where do educational innovations come from that correspond to the state-of-the-art not only with regard to the content but also with regard to educational and methodological aspects? Who advances the reform of medical curricula to prepare the graduates comprehensively and future-proof for clinical practice? Who qualifies medical teachers for their changing duties especially regarding competency-based medical education? How can we make sure that the evidence from educational research translates into evidence-based teaching and learning?

The Science Council mentions the subject of teaching only briefly, but unequivocally: “In medicine, as well as in other fields, targeted measures are required for the professionalization, quality development and quality assurance of teaching. They must be institutionally anchored at all university medical centers and be further developed on an evidence-based basis. To meet professional standards in teaching consideration must be given to teaching competencies when selecting faculty as well as to continuous faculty development for medical education and also to the systematic evaluation of teaching by the students.” (p. 42) [1].

But who is to move forward and develop the teaching in this required way? Wouldn´t it be reasonable to wish and to demand a structured career path for the clinically active physicians – the “Clinician Educator”? And how should such a position be defined? The “Clinician Educator” is not a new idea in the Anglo-American system [3]. But there too is struggle for the successful development of this career path [4] and there are a lot of different varieties at the medical schools. But certainly the “Clinician Educator” must not be confused with the teaching professors (“Lehrprofessoren”) or “lecturers” established at some medical faculties to teach 16 or 18 semester-week hours “away”.

It should be an option to already choose the "Clinician Scientist" career path during the first years of residency training and it should have a clearly defined profile of requirements and tasks that can relate to the coordination and innovation of teaching as well as to the didactic qualification of teachers. Besides a qualification as a medical fellow or a medical specialist a stepwise and mandatory qualification in medical education should include a Master of Medical Education (MME) degree. The number of such master programs is growing rapidly worldwide [5]. In the last 10 years more than 350 graduates of the master's programs in Switzerland and Germany have been qualified in the German-speaking community.

These “clinician educator” positions with a tenure option could be jointly financed by the respective clinic and the dean´s office or by an institute for medical education. Similar career paths are, of course, also desirable for the basic scientists in the form of a “Medical Science Educator”, since in these disciplines important questions arise especially regarding competency-based curricula, which require a corresponding methodological and educational expertise. If each faculty were to train one or two of these clinicians or medical science educators per year, this could contribute substantially and sustainably to the professionalization of teaching in German university medicine.

As an internationally visible open access journal the GMS Journal for Medical Education (JME) wants to contribute to this development as a platform for research and quality assurance in medical education.

It is about the fact that in German university medicine not only the research and the new generation of researchers are threatened. Teaching as a priority falls behind patient care and the freedom of research, which the Science Council has rightly called for. Teaching is inextricably connected with research – otherwise it would no longer be up-to-date und lead to stagnation. However, the career paths of “clinician” or “medical science educators” are by no means in contradiction, but underline the necessity of developing the all-important feature of teaching in the field of university medicine in contrast to pure research institutes or other hospitals of maximum care.

## Competing interests

The authors declare, that they have no competing interests.
